# Evaluation of daytime sleepiness and insomnia symptoms in OSA patients with a characterization of symptom-defined phenotypes and their involvement in depression comorbidity—a cross-sectional clinical study

**DOI:** 10.3389/fpsyt.2024.1303778

**Published:** 2024-03-01

**Authors:** Agata Gabryelska, Szymon Turkiewicz, Piotr Białasiewicz, Filip Grzybowski, Dominik Strzelecki, Marcin Sochal

**Affiliations:** ^1^ Department of Sleep Medicine and Metabolic Disorders, Medical University of Lodz, Lodz, Poland; ^2^ Department of Affective and Psychotic Disorders, Medical University of Lodz, Lodz, Poland

**Keywords:** insomnia, phenotyping, OSA, depression, excessive daytime sleepiness

## Abstract

**Introduction:**

Recent research highlights the significance of insomnia and sleepiness, shifting from obstructive sleep apnea (OSA) severity and sleep structure, in defining OSA phenotypes.

**Objectives:**

This study aimed to characterize insomnia and sleepiness associated with OSA phenotypes and assess their involvement in depression symptoms (DS) in OSA.

**Materials and methods:**

This cross-sectional, clinical study included 181 participants who underwent polysomnography (PSG) and filled out questionnaires, including the Epworth Sleepiness Scale (ESS), Insomnia Severity Index (ISI), Pittsburgh Sleep Quality Index (PSQI), and Beck Depression Index (BDI). They were categorized into phenotypes: insomnia**–**sleepiness (I + S; ESS ≥ 11; ISI ≥ 15; n = 20), sleepiness (S; ESS ≥ 11; ISI < 15; n = 22), insomnia (I; ESS < 11; ISI ≥ 15), and asymptomatic (A; ESS < 11; ISI<15; n=55).

**Results:**

A linear regression model for the BDI score (R^2^ = 0.357, p < 0.001) included ISI score and subjective-to-objective sleep latency ratio. The ISI score was a predictive factor for mild and moderate DS [OR = 1.23 (95% CI: 1.09–1.38), p < 0.001 and OR = 1.39 (95% CI: 1.13–1.72), p = 0.002]. The I and I + S phenotypes are characterized by higher BDI scores (p < 0.001 and p = 0.02), longer subjective sleep latency (p = 0.008 and p = 0.04), and shorter subjective total sleep time (TST; p = 0.049 and p = 0.006) compared to A. Furthermore, the I and I + S groups had shorter subjective TST than S (p = 0.03 and p = 0.047). The I and I + S had higher BDI scores than A (p < 0.001 and p = 0.02, respectively) and S (p < 0.001 and p = 0.02, respectively). The I phenotype was associated with the risk of mild and moderate DS (OR = 5.61 (95% CI: 1.91–16.53), p < 0.001 and OR = 9.55 (95% CI: 1.81–50.48), p = 0.008 respectively). Moreover, the I + S phenotype presented an even greater risk for mild DS (OR = 10.29 (95% CI: 2.95–35.85), p < 0.001).

**Conclusion:**

Using clinical features for OSA phenotyping holds promise for finding OSA individuals with increased risk for DS occurrence.

## 1 Introduction

Obstructive sleep apnea (OSA) is a notable sleep–breathing disorder marked by recurring episodes during sleep wherein the individual experiences partial or full upper airway collapse. These episodes bring about a discernible reduction (known as hypopnea) or a complete obstruction (apnea) of the upper airways limiting airflow (1). On the global scale, the influence of this condition extends to a substantial demographic impacting a population of approximately 936 million adults aged 30–69 years (2). The common risk factors for OSA include male sex, age 40–70 years, familial aggregation, body habitus (obesity/overweight, central body fat distribution, and large neck girth), or craniofacial and upper airway abnormalities (3).

The gold standard of OSA diagnostics is overnight polysomnography (PSG) assessment, which allows monitoring of various parameters of sleep. During the procedure, the number of apneas and hypopneas is calculated generating the Apnea–Hypopnea Index (AHI), which is used to diagnose and assess the severity of OSA (severe, moderate, or mild) (4).

The impact of OSA on the quality of life of an individual is unquestionable and supported by a diverse array of research studies demonstrating its influence on both physical and mental health. Among the comorbidities accompanying this condition, the prominent ones include, among others, metabolic syndrome (5), atherosclerosis (6), autoimmune disease (7), and depression (8). In recent years, greater focus has been put on psychiatric complications of OSA. Multiple symptoms overlap between OSA and depression with suggested, yet poorly understood, causal link (9). Furthermore, OSA is also associated with insomnia characterized by difficulty initiating or maintaining sleep (10) followed by daytime symptoms such as fatigue, mood disturbance, excessive sleepiness, and impairment of cognitive performance (11).

The practice of phenotyping is a prevalent strategy in various respiratory disorders, such as Chronic obstructive pulmonary disease (COPD) and asthma, and has been extensively embraced. The complexity observed in those conditions has spurred clinicians to categorize the symptomatology of patients into phenotypes (12). In OSA, those groups can be predicated on a spectrum of characteristics encompassing disease symptoms, quality of life, outcomes, or even genetic characteristics. Various phenotypes in OSA include, among others, Rapid eye movement phase of a sleep (REM) dependent, REM independent, positional, sleepiness, and comorbid insomnia in OSA (COMISA). The first one has two different definitions. REM phenotype OSA may be diagnosed when AHI during REM is at least twice as high as NREM OSA or AHI during REM is five or more, while Non-rapid eye movement phase of a sleep (NREM) AHI is less than five. Both definitions have a common requirement of a total REM duration of at least 30 min throughout the whole sleep (13). The REM-dependent OSA is related with cardiovascular and metabolic issues, for instance, hypertension (14), cardiac metabolic dysfunction (15), and diabetes mellites type 2 (DM2) (16). In contrast, the REM-independent phenotype is similar to general OSA. The overall AHI of those patients remains relatively stable during both REM and NREM sleep. Positional OSA is divided into two groups: supine-predominant and supine-isolated OSA. Supine-isolated OSA patients were characterized by not only lower arousal index but also poorer sleep quality, and more depressed and anxious compared to the supine-predominant group (17). Moreover, positional OSA may be treated through positional therapy (18).

The clinical manifestation of OSA seems to be the future way to define OSA phenotypes, such as sleepiness phenotype. Individuals with sleepiness OSA primarily present with excessive daytime sleepiness often associated with increased fatigue, a higher propensity to fall asleep during daytime activities (19), and OSA severity (20). Moreover, the sleepiness phenotype is associated with the REM-dependent OSA. Both were characterized by increased Epworth Sleepiness Scale (ESS) and similar comorbidities such as cardiovascular impairment (20).

Insomnia is another common symptom of OSA, and it is also under examination in the context of the OSA phenotype. For instance, COMISA is characterized by insomnia associated with prolonged awakenings resulting in increased sleep disturbance compared to that of patients with OSA but without insomnia (21). The insomnia phenotype is also related to an increased risk of psychiatric comorbidity development (20) including depression (22). Co-existence of insomnia and OSA is associated with lower continuous positive airway pressure (CPAP) adherence and response to the treatment compared to the sleepiness phenotype (20). However, if the CPAP treatment is effective, the insomnia symptoms are likely to improve (23).

Therefore, the aims of the study was to characterize insomnia and sleepiness associated with OSA phenotypes and to assess the involvement of the symptoms and phenotypes in the pronouncement of depression symptoms (DS) in OSA patients.

## 2 Materials and methods

### 2.1 Sample

**Figure 1:**
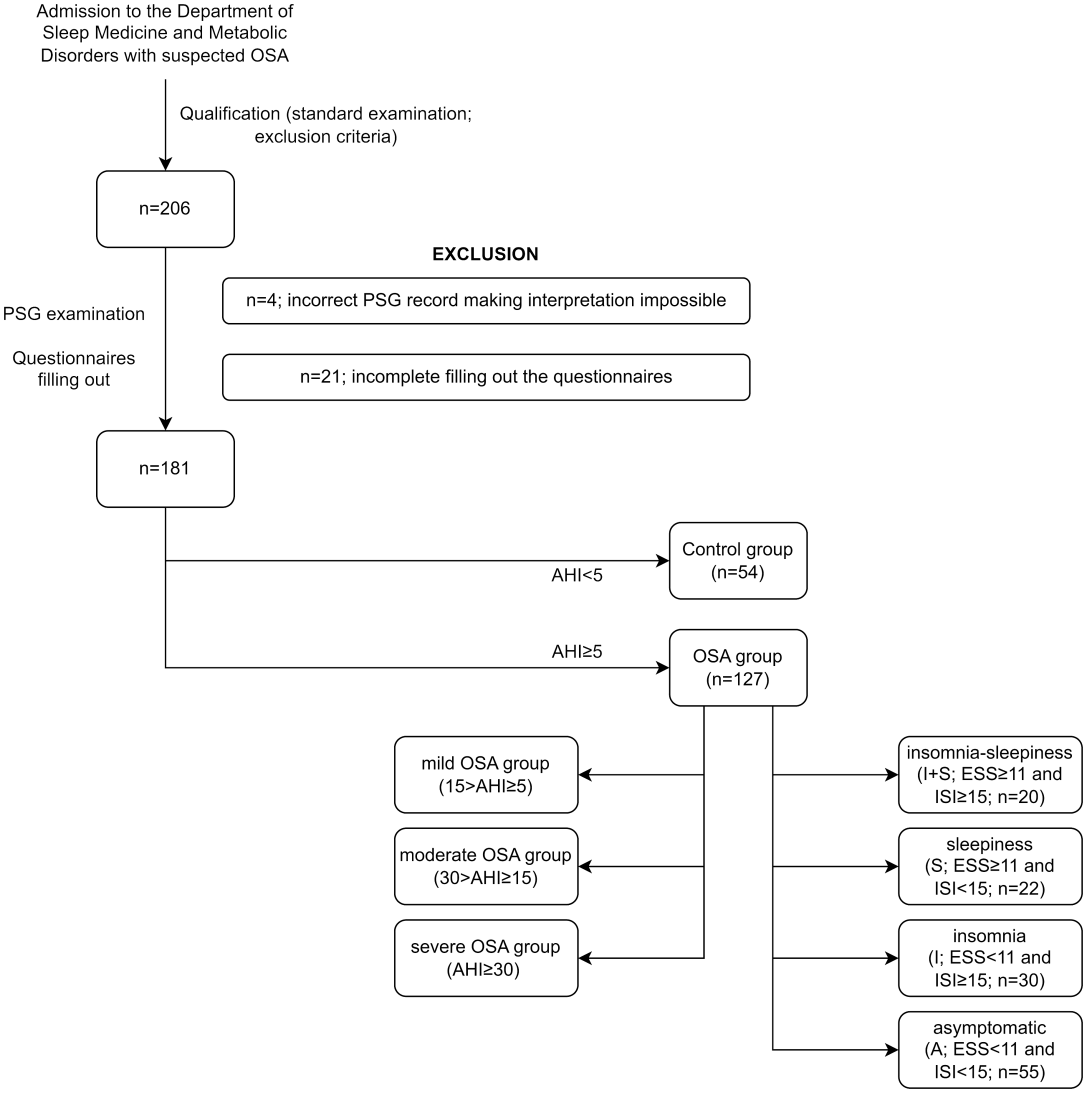
Study groups. AHI, Apnea–Hypopnea Index; ESS, Epworth Sleepiness Scale; ISI, Insomnia Severity Index; OSA, obstructive sleep apnea; PSG, polysomnography.

The study has a cross-sectional, clinical character and was conducted from July 2018 to September 2021. The study group consisted of 181 participants referred to the Sleep and Respiratory Disorders Center in Lodz (Poland) with a presumptive diagnosis of OSA (**Figure 1**). All participants underwent standard nocturnal polysomnography (PSG) examination. The exclusion criteria included inflammatory diseases (e.g., connective tissue diseases or inflammatory bowel diseases), chronic respiratory diseases (e.g., asthma or chronic obstructive pulmonary disease), diagnosis of cancer (active or in medical history), diagnosed major neurological conditions, diagnosed psychiatric disorders, and taking medications affecting sleep (e.g., benzodiazepines and melatonin). All the above data were collected from standard physical examination and available medical documentation. The study was approved by the Ethics Committee of the Medical University of Lodz (RNN/432/18/KE). All patients provided written informed consent to participate in the study.

### 2.2 Polysomnography

Participants were admitted to the sleep lab at 21:00 ( ± 0.5 h) and underwent physical examination (measurement of body mass, height, heart rate, and blood pressure) performed by a physician. The PSG examination room had a set temperature at 20°C. The time of examination was 9:00 (lights out: 21:00–21:30; lights on: 6:00–6:30). The following channels were used to perform nocturnal PSG: electroencephalography (C4\A1, C3\A2), chin muscles and anterior tibialis electromyography, electrooculography, measurements of oronasal airflow (a thermistor gauge), snoring, body position, respiratory movements of chest and abdomen (piezoelectric gauges), unipolar electrocardiogram and hemoglobin oxygen saturation (*SpO2*) (Alice 6, Phillips-Respironics). The criteria based on a 30-s epoch standard were used to score sleep stages in the recorded PSG (24). Scoring was performed by the same physician–researcher specializing in PSG evaluation. Apnea was defined as the reduction of airflow to less than 10% of the baseline for at least 10 s. Hypopnea was described as at least a 30% reduction of airflow for at least 10 s, accompanied by an over 3% decrease in *SpO2* or arousal. The American Academy of Sleep Medicine guidelines were used to score the arousals (24).

### 2.3 Questionnaires

Questionnaires used in the study included the following: the Insomnia Severity Index (ISI), Epworth Sleepiness Scale (ESS), Beck Depression Inventory (BDI), and Pittsburgh Sleep Quality Index (PSQI), and all were filled out by each participant in the morning following the PSG examination.

#### 2.3.1 Insomnia severity index (ISI)

A self-evaluation questionnaire was used to assess the severity of insomnia. Patients were asked to answer seven questions, which evaluate aspects of sleep patterns such as difficulty in falling or staying asleep, wakening up too early, dissatisfaction with sleep pattern, interference of sleep problems with daily functioning, sleep problem’s impact on quality-of-life noticeability, and worry/distress about sleep. Participants used a scale ranging from 0 to 4 to rate the extent to which each question applies to their condition. The answers were added up to get a general score. Based on this result, patients were categorized into four groups as follows: no clinically significant insomnia (0–7), subthreshold insomnia (8–14), moderate-severity clinical insomnia (15–21), and severe clinical insomnia (22–28).

#### 2.3.2 Pittsburgh sleep quality index (PSQI)

This is a self-evaluation questionnaire assessing seven various sleep aspects in adults. It evaluates sleep quality parameters such as difficulties with falling asleep, problems with maintaining continuity of sleep, functioning during the day, and questions regarding the most frequent causes of sleep disorders over the past 4 weeks. They all make up the outcome, assessed from 0 to 21 points. Results higher than 5 points indicate low sleep quality and differentiate patients as having “poor” and “good” sleep, with higher scores corresponding to poorer quality of sleep (25–27). Only questions related to subjective sleep latency (question 2) and total sleep time (question 4) were analyzed. Based on subjective sleep latency from PSQI (question 2) and objective sleep latency from PSG, the two following variables were created: subjective-to-objective sleep latency ratio presented in percentages and the difference between subjective and objective sleep latency presented in minutes. Similarly, based on subjective total sleep time from PSQI (question 4) and objective total sleep time from PSG, the subjective-to-objective total sleep time ratio presented in percentages was created as a variable (25–27). A validated PSQI version in Polish was used in the study (28).

#### 2.3.3 Beck depression inventory (BDI)

The self-evaluation questionnaire consists of 21 questions, which assess the intensification of each depression symptom on a four-grade scale from 0 to 3 points. All answers were added up, giving a maximum score of 63 points. The BDI interpretation divides results into four groups based on the intensity of DS as follows: slight depression(0–13), mild depression (14–19), moderate depression (20–28), and severe depression (29–63) (29). The questionnaire does not define a lack of depression. A validated BDI version in Polish was used in the study (30, 31).

#### 2.3.4 Epworth sleepiness scale (ESS)

The questionnaire consists of eight questions in which patients assess from 0 to 3 their likeness of falling asleep in a given situation (for instance, sitting and reading, watching tv, sitting and talking to someone). It is used to assess excessive daytime sleepiness. Cumulative scores are made from the summation of responses. Scores from 0 to 7 indicate a low likelihood of abnormal sleepiness. Those within the range of 8 to 9 exhibit an average level of daytime sleepiness. Individuals scoring between 10 and 15 might experience excessive sleepiness. In cases where scores range from 16 to 24, individuals demonstrate significant daytime sleepiness (32, 33).

### 2.4 Study groups

Based on the AHI from the PSG examination, patients were divided into the healthy control group (n = 54; AHI < 5) and the OSA group (n = 127; AHI ≥ 5), which was first subdivided into the mild OSA group (15 > AHI ≥ 5), moderate OSA group (30 > AHI ≥ 15), and the severe OSA group (AHI ≥ 30) and second into four symptom-based phenotypes, which were defined as insomnia–sleepiness (I + S; ESS ≥ 1 and ISI ≥ 15), sleepiness (S; ESS ≥ 11 and ISI < 15), insomnia (I; ESS < 11 and ISI ≥ 15), and asymptomatic (A; ESS < 11 and ISI ≥ 15) phenotypes.

### 2.5 Statistical analysis

The level of statistical significance was set at p < 0.05. Statistical analysis was performed with SPSS 28.0 (IBM, Chicago, IL, USA). The distribution of variables was evaluated by the Shapiro–Wilk test. The parameters with normally distributed data were compared by independent-sample t-test and one-way ANOVA test with *post hoc* Tukey tests. Normally distributed data are presented as mean ± standard deviation or median and interquartile range (IQR) to allow for comparison with other variables. Comparisons of parameters without normal distribution were performed by Mann–Whitney U and Kruskal–Wallis tests with *post-hoc* Dunn’s tests with results presented as the median and interquartile range (IQR). Chi-square, Chi-square tests with Yate’s correction, and Fisher test were used to assess nominal variables in situations where the size of the smallest group was above 15, in the range of 5–15, and below 5, respectively. Spearman’s rank correlation was used to assess correlations. For multiple testing, the Bonferroni correction was applied. Multivariable linear regression with a stepwise procedure was performed to analyze the investigated predictive factors of the BDI score. Logistic regression was performed to assess the odds ratio (OD) of chosen parameters as the risk of mild and moderate depression.

## 3 Results

Baseline characteristics including demographic data, PSG parameters, questionnaire results, and comparisons between distinct OSA phenotypes within the control and the OSA groups as well as within OSA severity subgroups are as follows: mild OSA, moderate OSA, and severe OSA are shown in **Supplementary Tables 1, 2**, respectively. The OSA group (n = 127) was characterized by a median age of 54.09 ( ± 11.47) years, a mean BMI of 27.13 (24.34–31.74), and 83.5% of the male sex. The control group (n = 54) was characterized by a median age of 45.87 ( ± 12.68), a mean BMI of 27.12 (24.34–31.81), and 64.8% of participants were males. The OSA group was divided based on OSA severity into mild OSA (n = 42), moderate OSA (n = 29), and severe OSA (n = 56).

The phenotypes within the OSA group and OSA severity groups were matched in demographic data in (p > 0.05) **Supplementary Tables 1, 2**, respectively.

Regarding PSG parameters, the only difference was observed in total sleep time (TST) between I + S and A groups with the former having longer TST (p = 0.029). Sleep latency was only longer subjectively in the I and I + S groups compared to that in the A group (p = 0.049 and p = 0.006, respectively). At the same time, no differences were observed in objective sleep latency between the phenotypes within the OSA group (p = 0.078). Moreover, both the I and I + S groups had longer subjective than objective sleep latency compared to the A group (p = 0.008 and p = 0.041, respectively). Furthermore, the I group had shorter subjective TST than the A (p = 0.005) and S (p = 0.028) groups; similarly, the I + S group had shorter subjective TST than the A (p = 0.016) and S (p = 0.047) groups.

Concerning the pronouncement of DS, the I and I + S groups had higher BDI scores than the A (p < 0.001 and 0.015, respectively) and S (p < 0.001 and p = 0.017, respectively) groups. Additionally, the frequency of individuals fulfilling the criteria for mild and moderate DS severity was greater in the I group than in the A (p = 0.002 and p = 0.003, respectively) and S groups (p = 0.042 and p = 0.025, respectively). Similarly, it was higher in the I + S group than in the A (p < 0.001 and p = 0.043, respectively) and S groups (p = 0.010 and p = 0.043, respectively). Furthermore, the I + S phenotype had a higher prevalence of severe DS than the A (p = 0.017) and S (p = 0.049) phenotypes.

In comparisons between the control and OSA groups as well as the OSA severity groups (**Supplementary Table 3**), no differences were observed in BDI scores, mild, moderate, and severe DS frequency either in full groups or within all phenotype subgroups.

In the OSA group, the BDI score increased with greater scores of the following questionnaire variables: ESS (r = 0.205, p = 0.021), ISI (r = 0.578, p < 0.001), PSQI Item 2 (r = 0.199, p = 0.025), and subjective-to-objective sleep latency ratio (r = 0.208, p = 0.019), while it was negatively correlated with PSQI Item 4 (r = −0.203, p = 0.022). Furthermore, BDI was positively associated with the following PSG parameters: AHI (r = 0.233, p = 0.012), AHI in REM (r = 0.194, p = 0.031), AHI in NREM (r = 0.246, p = 0.012), and Desaturation Index (r = 0.210, p = 0.018). Last, from the demographic parameters, a positive correlation was observed between the BDI score and BMI (r = 0.207, p = 0.020).

Among the OSA participants, a multivariable linear regression was constructed for the BDI score by stepwise elimination. The model explained 35.7% of the variance (p < 0.001) and included the following parameters: ISI score (B = 0.743, p < 0.001), subjective-to-objective sleep latency ratio (B = 0.001, p = 0.049), and BMI (B =−0.146, p = 0.134) (**Table 1**).

**Table 1:** Linear regression model for BDI score.

BDI score				
Model	R^2^ = 0.357, F = 23.546, p < 0.001			
Parameters	B	B 95% CI	t	p-value
Constant	−3.779	−10.376–2.818	−1.134	0.259
**ISI score**	**0.743**	**0.527–0.960**	**6.801**	**<.001**
**Subjective to Objective Sleep Latency Ratio**	**0.001**	**0.000–0.003**	**1.988**	**0.049**
BMI	0.146	−0.046–0.337	1.508	0.134

AHI, Apnea–Hypopnea Index; BDI, Beck Depression Index; BMI, body mass index; CI, confidence interval; ESS, Epworth Sleepiness Scale; ISI, Insomnia Severity Index; PSQI, Pittsburgh Sleep Quality Index. Bold values are those that were statistically significant.

Excluded variables: ESS, PSQI Item 4 score, AHI, Desaturation Index, PSQI Item 2 score.

Next, logistic regression models were generated to evaluate the involvement of the chosen parameters in the presence of mild (BDI score ≥ 14) and moderate (BDI score ≥ 20) DS. For the occurrence of severe DS mild (BDI score ≥ 29) model was not constructed due to an insufficient number of individuals achieving a high-enough BDI score.

The ISI score was the only significant risk factor for the presence of both mild and moderate DS. With one one-point increase in the ISI score, the risk for mild DS increased 1.23 times (95% CI: 1.09–1.38, p < 0.001), while for moderate DS, the risk increased 1.39 times (95% CI: 1.13–1.72, p = 0.002). No other questionnaire, demographic, or PSG variables were found to increase the risk for the occurrence of mild and moderate DS. Full data for the evaluation of risk factors for mild and moderate DS are presented in **Table 2**.

**Table 2:** Logistic regression for mild and moderate depression cut-offs for BDI scores including questionnaire, PSG, and demographic data.

		BDI score ≥ 14 (mild depression symptoms)				BDI score ≥ 20 (moderate depression symptoms)			
		B	OR	OR 95% CI	*p*-value	B	OR	OR 95% CI	*p*-value
Parameters	ESS score	0.029	1.03	0.93–1.14	0.589	−0.067	0.94	0.81–1.08	0.377
	**ISI score**	**0.204**	**1.23**	**1.09–1.38**	**<0.001**	**0.331**	**1.39**	**1.13–1.72**	**0.002**
	Subjective sleep latency (PSQI Item 2)	0.002	1.00	0.98–1.02	0.852	−0.009	0.99	0.96–1.02	0.552
	Subjective-to-objective sleep latency ratio	0.000	1.00	1.00–1.00	0.755	0.000	1.00	1.00–1.00	0.556
	Subjective total sleep time (PSQI Item 4 score)	−0.112	0.89	0.63–1.27	0.533	−0.092	0.91	0.51–1.63	0.756
	BMI	0.079	1.08	0.97–1.21	0.156	0.007	1.01	0.85–1.20	0.932
	AHI	−0.032	0.97	0.90–1.04	0.396	0.001	1.00	0.88–1.13	0.991
	Desaturation Index	0.022	1.02	0.95–1.10	0.564	0.000	1.00	0.88–1.14	1.000
	**Constant**	**−5.622**	**0.004**	**–**	**0.011**	**−6.130**	**0.002**	**–**	**0.077**
Hosmer and Lemeshow test		8.639 (p = 0.374)				5.722 (p = 0.678)			
Pseudo-R^2^	Nagelkerke	0.315				0.373			
	McFadden	0.207				0.296			

AHI, Apnea–Hypopnea Index; BDI, Beck Depression Index; BMI, body mass index; ESS, Epworth Sleepiness Scale; ISI, Insomnia Severity Index. Bold values are those that were statistically significant.

Furthermore, logistic regression models were created to evaluate the involvement of the studied OSA phenotypes in the occurrence of mild and moderate DS. The insomnia phenotype increased the risk of mild and moderate presence 5.61 (95% CI: 1.91–15.53, p < 0.001) and 9.55 (95% CI: 1.81–50.48, p = 0.008) times respectively. In addition, the risk of mild DS occurring was 10.29 (95% CI: 2.95–35.85, p < 0.001) times higher among OSA patients with I + S phenotype compared to asymptomatic individuals. The sleepiness phenotype did not affect the occurrence of mild DS (p = 0.731), while the assessment was not performed for moderate DS as no individuals fulfilled the criteria. Full data for the assessment of OSA phenotype involvement in mild and moderate DS presence are presented in **Table 3**.

**Table 3:** Logistic regression for mild and moderate depression cut-offs for BDI scores including phenotypes, PSG, and demographic data.

		BDI score ≥ 14 (mild depression symptoms)				BDI score ≥ 20 (Moderate depression symptoms)			
		B	OR	OR 95% CI	p-value	B	OR	OR 95% CI	p-value
Parameters	BMI	0.074	1.08	0.97–1.20	0.166	0.056	1.06	0.91–1.23	0.460
	AHI	−0.028	0.97	0.91–1.04	0.425	0.020	1.02	0.92–1.13	0.702
	Desaturation Index	0.019	1.02	0.95–1.09	0.593	−0.015	0.99	0.89–1.10	0.784
	**Phenotype**	**-**	**-**	**-**	**<0.001**	**-**	**-**	**-**	**0.040**
	**Phenotype insomnia**	**1.725**	**5.61**	**1.91–16.53**	**0.002**	**2.257**	**9.55**	**1.81–50.48**	**0.008**
	Phenotype sleepiness	0.239	1.27	0.33–4.96	0.731	-	-	-	-
	**Phenotype insomnia ± sleepiness**	**2.331**	**10.29**	**2.95–35.85**	**<0.001**	**1.759**	**5.81**	**0.91–37.13**	**0.063**
	**Constant**	**−3.914**	**0.02**	**-**	**0.015**	**−5.225**	**0.005**	**-**	**0.026**
Hosmer and Lemeshow test		11.819 (p = 0.159)				4.441 (p = 0.815)			
Pseudo-R^2^ Nagelkerke		0.253				0.269			
Omnibus tests of model coefficients		24.676 (p < 0.001)				18.202 (p = 0.006)			

AHI, Apnea–Hypopnea Index; BDI, Beck Depression Index; BMI, body mass index. Bold values are those that were statistically significant.

## 4 Discussion

In this study, we focused on symptom-defined phenotypes, such as insomnia or daytime sleepiness, which are commonly presented by OSA patients (34).

The main aim of the study was to assess the OSA phenotypes based on their clinical features. We created four phenotypes based on ISI and ESS scores (I, S, I + S, and A) and checked the possible connection with the severity of DS measured by BDI. As a result, we found that the insomnia phenotype of OSA predisposes to an increase in DS severity and development. In addition, excessive daytime sleepiness, which is another common symptom of OSA, has an impact on this association only in the situation of co-existence with insomnia.

OSA has a complex clinical presentation; its manifestation overlaps with depression, including lack of energy, increased fatigue, or cognitive impairment (35). Recent studies showed that both disorders are associated with each other, and the insomnia phenotype of OSA is characterized by an increased risk of psychiatric comorbidity development (20, 35), which is in line with our results. This shows a great need to further investigate the association and possible underlying mechanisms.

In our study, we found no differences in the severity of DS between the OSA and the control group or between the OSA severity groups. Available data on the topic are inconsistent. While some studies did not reveal differences between healthy and OSA individuals (10, 36), others suggest greater prevalence of DS in the OSA population (37, 38). The relationship between the severity of OSA, namely, AHI, and the severity of DS is also complicated (38). Lee et al. observed a correlation between the severity of OSA and DS (39). This study showed that the severity of DS in OSA patients increases with OSA severity and other PSG parameters, including AHI, AHI in REM, AHI in NREM, and Desaturation Index. It could indicate the possible impact of intermittent hypoxia on depression development, which is the baseline mechanism of oxidative stress and inflammation in the OSA (40). Microglia in brain tissue, in response to oxidative damage, may activate NF-κβ (nuclear factor κβ) and AP-1 (activator protein-1)-dependent pathways leading to neuroinflammation (41). This process may be aggravated by adrenergic overactivity in OSA patients (41). Moreover, intermittent hypoxia in OSA is related to the overactivity of hypoxia-inducible factor 1 (HIF-1), which is the main molecule intermediary between the hypoxia state and hypoxia-dependent response (42). OSA patients are characterized by overexpression of oxygen-dependent alpha subunit of HIF-1 (43, 44). There is some evidence that HIF-1 alpha may have an impact on the brain-derived neurotrophic factor (BDNF) expression (8), whose pathway disturbance is widely investigated in the context of depression development (discussed in more detail in later sections). Nevertheless, a high discrepancy in the outcomes suggests a possible need to find other methods of OSA phenotyping other than just severity as well as a complex pathomechanism and probable heterogeneity.

Considering that we found that the severity of DS is positively correlated with insomnia and excessive daytime sleepiness, we decided to define OSA phenotypes based on both OSA symptoms. The study showed that ISI scores are a predicting factor for greater BDI scores corresponding to mild or moderate depression. Additionally, it was revealed that the I phenotype notably increases the risk of developing mild and moderate DS. Moreover, the co-occurrence of insomnia and excessive daytime sleepiness results in an even greater risk. Oppositely, in the S phenotype, there is no similar association. OSA, as a state of systemic inflammation, is correlated with many comorbidities. Saaresranra et al. showed, in the study on 6,555 Israelites, that the insomnia phenotype of OSA was correlated with an increased risk of cardiovascular and psychiatric comorbidity, which was independent of age, gender, and BMI (20). Interestingly, it was observed that OSA severity was associated only with excessive daytime sleepiness phenotype, not with the insomnia one (20). In the study by Frangopoulos, the symptomatic group (insomnia + sleepiness, measured by the Athens Insomnia Scale and ESS, respectively) was marked by increased anxiety, fatigue, depression, and poor sleep quality (45). All the above studies showed just a connection between insomnia and symptom severity in OSA, but they did not explain the mechanism. Zhang et al. studied the genetic background of the OSA and tried to connect its clinical phenotypes with cognitive aging. They found that the Genetic Risk Score (an estimate of the cumulative contribution of genetic factors to a specific outcome of interest in an individual) for insomnia and daytime sleepiness are associated with an increased risk of mild cognitive impairment development and decreased global cognitive function (46). Despite genetics and inflammation, outcomes of recent studies indicate also the possible impact of the BDNF signaling pathway on the association of insomnia and depression in the OSA. It has been observed that serum BDNF and proBDNF evening levels (before PSG) were higher in the OSA group with elevated severity of DS and lower in the morning (after PSG) (10). BDNF and its premature form are neurotrophins whose main function is to regulate the function and survival of the neurons (8). Their pathway disturbance has been associated already with neurocognitive decline, sleep disturbances, and depression; hence, it is a convincing argument to consider its possible role in the pathophysiology of depression and insomnia in OSA patients (8). Moreover, the differences in the evening and morning protein levels (before and after PSG) point out the possible impact of the circadian clock on the BDNF signaling pathway. A more in-depth evaluation is needed to fully understand the underlying mechanisms of complex relationships between symptomatology, OSA itself, and DS.

In addition, our study revealed that phenotypes with insomnia were characterized by increased objective and subjective sleep latency (PSQI Item 2) and subjective total sleep time (PSQI Item 4). Moreover, the severity of DS was positively correlated with subjective sleep latency, subjective-to-objective sleep latency ratio, and negatively with subjective total sleep time. Interestingly, objective total sleep time was longer in the I + S phenotype compared to those in other phenotypes. It is established that insomnia disorder is related to subjective features such as sleep-onset latency (SOL) and total sleep time. In contrast, OSA was associated mainly with objective parameters such as longer total sleep time and shorter SOL (47). Zhuang et al. also pointed out that subjective sleep perception of OSA patients was mainly associated with sleep structure and respiratory events, and that of insomnia patients, to sleep latency. In our study, extended subjective and objective sleep latency characterized the insomnia phenotype of OSA. This indicates that the characteristic symptoms associated with insomnia resemble OSA. Interestingly, other researchers noticed a similar relationship. For example, in the study by Ma et al., self-reported insomnia was linked to discrepancies in sleep and a diminished quality of life. Individuals with both insomnia and comorbid OSA experience the most notable sleep discrepancy and report the lowest quality of life (48). This is further supported by the fact that insomnia-associated phenotypes (I and I + S) were characterized by underestimation of total sleep time observed in comparisons of subjective-to-objective sleep ratios. Furthermore, we showed that also DS correlated with subjective features of sleep discrepancy. It emphasizes the connection between the severity of depression and insomnia manifestation and underscores the importance of understanding how different sleep disorders, such as insomnia and OSA, influence both objective sleep parameters and subjective sleep perception ultimately impacting the quality of life of an individual. This highlights a subjective aspect of insomnia as a disorder, regardless of co-existence of other sleep disorders, in this case, OSA (49).

Some limitations of the study should be mentioned. First and the main limitation of our study was the small size of some subgroups, which restricted statistical analysis preventing the inclusion of the severe DS group. Second, insomnia and depression assessments were based on questionnaires with no other clinical investigation. Furthermore, it is important to note that the cross-sectional design of the study limits our ability to determine causality. Additionally, some confounding variables, such as anxiety levels or hormone concentrations, were not measured but could have an effect on insomnia and depression severity. To fully understand the nature of this complex association, prospective, interventional trials are needed, with inclusion of both symptoms and PSG defined phenotypes.

## 5 Conclusion

The findings of this study shed light on the complex interplay between OSA and DS. The insomnia phenotype of OSA significantly predisposes individuals to a presence and an escalation in DS severity. Furthermore, excessive daytime sleepiness, a common symptom of OSA, appears to influence this relationship when both symptoms coexist, underscoring the multifaceted nature of OSA. Phenotyping OSA using clinical manifestations, such as insomnia and excessive daytime sleepiness, is essential as it enables a more individualized and precise approach to the diagnosis, management, and treatment of OSA.

## Data availability statement

The raw data supporting the conclusions of this article will be made available by the authors, without undue reservation.

## Ethics statement

The studies involving humans were approved by Ethics Committee of the Medical University of Lodz (RNN/432/18/KE). The studies were conducted in accordance with the local legislation and institutional requirements. The participants provided their written informed consent to participate in this study.

## Author contributions

AG: Conceptualization, Formal analysis, Funding acquisition, Investigation, Methodology, Visualization, Writing – original draft, Writing – review & editing. ST: Investigation, Visualization, Writing – original draft, Writing – review & editing. PB: Investigation, Writing – review & editing. FG: Investigation, Writing – original draft. DS: Writing – review & editing. MS: Investigation, Methodology, Writing – review & editing.
