# Supplementary material for: Evaluation of daytime sleepiness and insomnia symptoms in OSA patients with a characterization of symptom-defined phenotypes and their involvement in depression comorbidity—a cross-sectional clinical study
Source: Front Psychiatry. 2024 Mar 1;15:1303778. doi: 10.3389/fpsyt.2024.1303778 (PMC10940440; doi:10.3389/fpsyt.2024.1303778)
Supplement: Supplementary file 1 [file Table_1.docx]

Supplementary Material

Evaluation of daytime sleepiness and insomnia symptoms in OSA patients with a characterization of symptom-defined phenotypes and their involvement in depression comorbidity – a cross-sectional clinical study

**Agata Gabryelska*, Szymon Turkiewicz, Piotr Białasiewicz, Filip Grzybowski, Dominik Strzelecki, Marcin Sochal**

*** Correspondence:** Agata Gabryelska, MD, PhD; Department of Sleep Medicine and Metabolic Disorders at the Medical University of Lodz, 6/8 Mazowiecka Street, 90-419 Lodz, Poland, tel. +48 422725660, e-mail: agata.gabryelska@gmail.com

# Supplementary Table 1: Baseline characteristics and comparisons between distinct OSA phenotypes within the control and the OSA groups.

|  | | Control Group | | | | | | OSA Group | | | | | |
| --- | --- | --- | --- | --- | --- | --- | --- | --- | --- | --- | --- | --- | --- |
| Phenotype | | Full Group | Asymptomatic | Insomnia | Sleepiness | Insomnia +Sleepiness | p-value* | Full Group | Asymptomatic | Insomnia | Sleepiness | Insomnia +Sleepiness | p-value* |
| Demographic Data | N | 54 | 23 | 13 | 7 | 11 | N/A | 127 | 55 | 30 | 22 | 20 | N/A |
|  | Age [years] | 45.87 +/- 12.68 | 48.61 +/- 10.83 | 48.69 +/- 1.82 | 39.57 +/- 12.93 | 40.82 +/- 14.58 | 0.188 | 54.09 +/- 11.47 | 55.69 +/- 11.71 | 53.15 +/- 10.23 | 56.23 +/- 11.50 | 48.75 +/- 11.50 | 0.119 |
|  | BMI [kg/m2] | 27.13 (24.34 - 31.81) | 26.30 (24.34-31.74) | 28.72 (24.81-34.07) | 28.34 (26.78-31.89) | 25.61 (23.63-34.69) | 0.638 | 30.86 (27.61-35.96) | 30.46 (26.98-34.63) | 31.87 (28.03-35.12) | 31.87 (26.96-36.74) | 31.67 (28.91-39.06) | 0.629 |
|  | Sex (M) [%] | 35 (64.8%) | 15 (65.2%) | 7 (53.8%) | 5 (71.4%) | 8 (72.7%) | 0.771 | 106 (83.5%) | 48 (87.3%) | 24 (80.0%) | 20 (90.9%) | 14 (70.0%) | 0.211 |
| PSG Data | Sleep Efficiency [%] | 83.80 (70.43 - 89.40) | 83.20 (67.48-89.40) | 77.40 (66.55-84.88) | 88.80(77.80- 95.80) | 85.90(65.00- 94.60) | 0.564 | 83.10 (74.53-90.10) | 79.80 (73.20-89.90) | 85.10 (72.30-90.85) | 79.90 (64.25-86.90) | 90.40 (83.10-92.90) | 0.105 |
|  | Sleep Onset Latency [min] | 24.25 (11.50 - 35.88) | 27.00 (13.00-43.50) | 34.50 (18.50-50.00) | 16.00 (7.50-30.00) | 11.0 (7.00-22.25) | 0.059 | 19.25 (10.00-31.50) | 24.50 (13.00-38.50) | 15.75 (7.50-29.38) | 19.00 (10.38-26.00) | 10.00 (6.50-26.00) | 0.078 |
|  | Sleep Maintenance Efficency [%] | 89.60 (82.30 - 96.75) | 90.05 (75.58-96.73) | 84.20 (69.00-96.80) | 92.30 (82.80-96.90) | 88.60 (84.70-96.70) | 0.778 | 89.45 (82.38-93.68) | 87.80 (82.30-93.90) | 90.50 (77.20-94.40) | 85.20 (68.75-95.00) | 91.70 (85.75-94.50) | 0.564 |
|  | REM Sleep Latency [min] | 103.50 (79.13 - 153.75) | 98.50 (73.50-158.00) | 128.00 (84.50-157.50) | 103.00(87.50-177.00) | 130.50 (77.0-151.50) | 0.755 | 89.75 (67.00-148.50) | 87.50 (65.00-152.50) | 86.50 (68.50-145.70) | 107.25 (58.25-153.75) | 87.50 (75.00-137.00) | 0.974 |
|  | Total Sleep Time (TST) [hours] | 6.20 (5.48 - 7.17) | 6.00 (5.30-6.50) | 6.37 (5.21-6.60) | 6.80 (5.20-7.50) | 6.60 (5.87-7.60) | 0.376 | 6.34 (5.40-6.97) | 6.17 (5.30-6.70) | 6.40 (5.42-7.04) | 6.25 (5.15-7.13) | 7.00 (6.07-7.63) | **0.047**  **0.029 ^c^** |
|  | REM Percentage of TST [%] | 21.15 (14.45 - 25.50) | 22.50 (18.30-26.30) | 18.00 (15.45-20.90) | 21.80 (10.40- 29.70) | 21.40 (12.80-24.70) | 0.369 | 19.90 (15.30-25.38) | 19.00 (15.30-23.20) | 21.95 (15.78-25.03) | 23.00 (12.00-28.75) | 19.70 (16.10-24.60) | 0.531 |
|  | Stage 1 Percentage of TST [%] | 14.10 (7.60 - 21.58) | 12.50 (5.20-21.70) | 17.70 (9.10-27.50) | 18.80 (10.20- 21.90) | 12.8 (7.90-17.55) | 0.411 | 29.55 (17.95-44.15) | 28.70 (16.60-44.00) | 33.90 (17.90-43.00) | 29.60 (18.33-57.23) | 24.00 (20.90-47.30) | 0.656 |
|  | Stage 2 Percentage of TST [%] | 43.80 (37.50 - 50.50) | 40.60 (36.90 - 49.10) | 48.80 (43.40 - 56.30) | 37.80 (35.00 - 46.70) | 43.20 (39.05 - 50.20) | 0.183 | 32.30 (24.93 - 43.80) | 35.60 (28.60 - 48.80) | 32.05 (21.33 - 43.83) | 25.75 (15.60 - 36.95) | 32.00 (25.80 - 41.00) | 0.053 |
|  | Stage 3 Percentage of TST [%] | 18.50 (12.00 - 25.95) | 22.60 (12.00-29.30) | 13.40 (9.50-22.35) | 18.20 (13.80-24.90) | 18.70 (11.85-29.35) | 0.570 | 13.75 (6.10-21.08) | 15.30 (6.20-19.30) | 13.20 (7.05-22.55) | 11.60 (0.78-20.73) | 13.90 (8.80-22.60) | 0.656 |
|  | REM Sleep Time [hours] | 1.25 (0.75 - 1.61) | 1.33 (1.01 - 1.68) | 1.11 (0.83 - 1.34) | 1.48 (0.44 - 2.05) | 0.92 (0.69 - 1.69) | 0.359 | 1.22 (1.66 - 0.82) | 1.14 (0.82 - 1.55) | 1.31 (0.84 - 1.80) | 1.25 (0.75 - 1.90) | 1.32 (0.86 - 1.62) | 0.635 |
|  | Stage 1 Time [hours] | 0.92 (0.41 - 1.34) | 0.76 (0.30-1.31) | 1.06 (0.56-1.50) | 1.17 (0.69-1.64) | 0.59 (0.12-1.075) | 0.195 | 1.80 (1.15-2.51) | 1.64 (1.07 +/-2.41) | 2.03 (1.07-2.52) | 1.87 (1.35-1.67) | 1.61 (1.30-2.62) | 0.564 |
|  | Stage 2 Time [hours] | 2.69 (2.17 - 3.28) | 2.54 (1.95 - 3.14) | 3.25 (2.47 - 3.73) | 2.35 (2.28 - 3.18) | 2.41 (1.72 - 3.18) | 0.355 | 2.04 (1.38 - 2.75) | 2.22 (1.65 - 3.18) | 2.01 (1.37 - 2.73) | 1.47 (1.00 - 2.42) | 2.13 (1.33 - 2.80) | 0.071 |
|  | Stage 3 Time [hours] | 1.00 (0.75 - 1.59) | 1.20 (0.76 - 1.64) | 0.86 (0.63 - 1.11) | 1.04 (0.95 - 1.45) | 1.01 (0.00 - 1.58) | 0.500 | 0.84 (0.37 - 1.25) | 0.96 (0.39 - 1.17) | 0.80 (0.40 - 1.38) | 0.77 (0.04 - 1.25) | 0.89 (0.46 - 1.43) | 0.697 |
|  | NREM Sleep Time [hours] | 4.90 (4.41 - 5.67) | 4.62 (4.13-5.44) | 5.12 (4.15-5.78) | 4.82 (4.36-5.45) | 5.51 (4.75-6.21) | 0.137 | 4.93 (4.27-5.48) | 4.85 (4.27-5.29) | 4.89 (4.32-5.58) | 4.92 (4.09-5.48) | 5.29 (4.52-6.29) | 0.236 |
|  | NREM Percentage of TST [%] | 79.15 (75.30 - 86.15) | 77.50 (73.70-81.70) | 82.00 (79.10-84.55) | 78.20 (70.30-89.60) | 86.10 (76.70-88.90) | 0.207 | 80.10 (74.80-84.70) | 81.00 (76.80-84.70) | 78.05 (74.96-84.23) | 77.00 (71.25-88.00) | 80.40 (75.58-84.95) | 0.538 |
|  | Arousal Index [events/hour] | 9.30 (5.00 - 13.10) | 9.20 (4.40-13.10) | 9.90 (7.45-15.15) | 11.90 (6.00-20.60) | 6.55 (3.30-9.13) | 0.258 | 17.50 (11.05-24.90) | 17.50 (10.90-23.20) | 18.15 (10.28-27.40) | 19.60 (11.55-33.60) | 16.20 (9.80-24.40) | 0.762 |
|  | AHI in REM [events/hour] | 2.69 (0.00 - 5.20) | 3.09 (0.00-5.02) | 3.33 (0.22-7.90) | 2.17 (0.00-4.88) | 2.42 (0.66-5.39) | 0.852 | 27.23 (9.47-46.78) | 22.85 (8.68-39.80) | 31.25 (12.20-51.36) | 30.71 (12.48-53.56) | 29.66 (15.84-53.84) | 0.306 |
|  | AHI in NREM [events/hour] | 1.30 (0.70 - 3.06) | 1.25 (0.72-2.53) | 1.20 (0.62-4.49) | 1.87 (0.83-3.67) | 1.29 (0.00-3.29) | 0.869 | 20.93 (9.98-43.53) | 19.48 (8.08-36.18) | 25.81 (10.32-42.99) | 20.00 (11.68-49.44) | 35.16 (14.27-51.76) | 0.391 |
|  | AHI [events/hour] | 1.80 (1.00 - 3.15) | 1.50 (1.00-2.85) | 2.20 (1.00-3.90) | 2.10 (0.90-3.90) | 1.60 (0.90-3.45) | 0.832 | 25.10 (12.00-46.80) | 20.30 (8.90-40.00) | 30.65 (10.48-51.55) | 25.00 (13.43-50.50) | 35.80 (13.20-52.00) | 0.204 |
|  | Total Number of Desaturations | 12.00 (6.00 - 23.00) | 10.00 (6.00-15.75) | 16.00 (5.00-24.00) | 26.00 (11.00-33.00) | 7.0 (1.50-20.50) | 0.126 | 147.00 (68.00-295.75) | 126.00 (56.00-235.00) | 211.00 (80.00-346.00) | 167.00 (65.50-329.50) | 176.00 (92.00-447.00) | 0.392 |
|  | Desaturation Index [events/hour] | 2.00 (1.00 - 3.00) | 2.00 (1.00-3.00) | 2.50 (1.00-3.95) | 3.00 (2.00-4.00) | 1.00 (00.00-4.00) | 0.138 | 26.00 (11.20-49.00) | 20.00 (8.10-39.70) | 32.15 (11.23-51.85) | 29.35 (13.50-58.75) | 31.65 (13.65-59.80) | 0.077 |
|  | Basal SpO_2_ [%] | 93.75 (92.68 - 95.025) | 93.90 (93.00-94.60) | 94.60 (91.95-95.70) | 94.50 (91.80-95.10) | 93.30 (93.00-95.30) | 0.904 | 92.50 (91.00-94.00) | 93.00 (92.00-94.00) | 92.05 (90.88-93.90) | 92.15 (90.00-93.38) | 92.10 (90.00-94.00) | 0.219 |
|  | Mean SpO_2_ during desaturations, [%] | 90.35 (88.80 - 92.45) | 90.10 (88.80-92.60) | 91.00 (88.40-93.00) | 90.90 (87.90-92.70) | 90.90 (88.90-92.00) | 0.889 | 88.30 (86.00-90.00) | 89.00 (87.10-90.00) | 88.00 (86.00-89.20) | 87.35 (82.75-89.00) | 87.95 (84.33-90.00) | 0.080 |
|  | Minimum SpO2 [%] | 87.90 (83.00 - 90.90) | 88.60 (85.90-90.90) | 85.00 (79.15-91.75) | 88.90 (83.00-91.90) | 86.95 (69.13-89.65) | 0.838 | 80.00 (73.95-84.00) | 80.70 (75.68-85.55) | 78.15 (73.18-82.03) | 76.40 (67.10-84.00) | 80.45 (66.58-82.75) | 0.333 |
| Questionaire Data | ESS score | 9.00 (5.00 - 11.25) | 6.00 (3.00-8.00) | 8.00 (4.50-9.00) | 12.00 (11.00-14.00) | 12.00 (11.00-13.00) | **<0.001**  **<0.0001^b^ <0.0001^c^ 0.005^d^ 0.001^e^** | 8.00 (5.00-12.00) | 6.00 (3.00-8.00) | 7.00 (4.00-9.00) | 12.50 (11.00-14.00) | 15.00 (12.00-17.00) | **<0.0001**  **<0.0001^a^**  **<0.0001^b^**  **<0.0001^c^**  **<0.0001^d^**  **<0.0001^e^** |
|  | ESS score $\geq$11 | 18 (33.3%) | 0 (0%) | 0 (0%) | 7 (100%) | 11 (100%) | **<0.0001**  **<0.0001^b^**  **<0.0001^c^**  **<0.0001^d^**  **<0.0001^e^** | 42 (33.1%) | 0 (0%) | 0 (0%) | 22 (100%) | 20 (100%) | **<0.0001**  **<0.0001^b^**  **<0.0001^c^**  **<0.0001^d^**  **<0.0001^e^** |
|  | ISI score | 14.00 (10.75 - 18.25) | 10.00 (7.00-13.00) | 18.00 (15.00-20.50) | 11.00 (11.00-14.00) | 21.00 (19.00-23.00) | **<0.001**  **<0.001^a^ <0.001^c^ 0.037^d^ 0.003^f^** | 12.00 (9.00-17.00) | 10.00 (7.00-12.00) | 17.00 (16.00-19.00) | 10.00 (8.00-12.00) | 17.00 (15.25-19.00) | **<0.0001**  **<0.0001**  **<0.0001^a^**  **<0.0001^b^**  **<0.0001^c^**  **<0.0001^d^**  **<0.0001^f^** |
|  | ISI score$\geq$15 | 24 (44.4%) | 0 (0%) | 13 (100%) | 0 (0%) | 11 (100%) | **<0.0001**  **<0.0001^a^**  **<0.0001^c^**  **<0.0001^d^**  **<0.0001^f^** | 50 (39.4%) | 0 (0%) | 30 (100%) | 0 (0%) | 20 (100%) | **<0.0001**  **<0.0001^a^**  **<0.0001^c^**  **<0.0001^d^**  **<0.0001^f^** |
|  | Subjective Sleep Latency (PSQI Item 2) [minutes] | 20.00 (12.50 - 60.00) | 15.00 (10.00-20.00) | 40.00 (26.25-60.00) | 10.00 (5.00-15.00) | 60.00 (37.50-75.00) | **<0.001**  **0.011^a^ 0.003^c^ 0.034^d^ 0.011^f^** | 20.00 (10.00-35.00) | 20.00 (10.00-30.00) | 30.00 (16.88-60.00) | 12.50 (7.13-20.75) | 17.50 (7.25-33.75) | **0.008**  **0.049^a^**  **0.006^d^** |
|  | Subjective to Objective Sleep Latency Ratio [%] | 97.69 (46.39 - 269.20) | 47.62 (28.57-142.86) | 102.27 (72.93-265.67) | 58.82 (47.62-375.00) | 342.86(202.49-640.90) | **0.001**  **0.001^c^** | 90.91 (49.59-235.71) | 76.92 (37.04-142.86) | 210.53 (80.10-427.48) | 61.25 (49.22-115.17) | 113.21 (25.00-300.00) | **0.002**  **0.004^a^**  **0.009^d^** |
|  | Difference between Subjective and Objective Sleep Latency [min] | 0.25 (-20.88 - 25.75) | -17.00 (-25.00-6.00) | 2.00 (-15.00-31.25) | -6.00 (-15.00-11.00) | 42.0 (15.00-50.00) | **<0.001**  **<0.0001^c^** | -0.50 (-11.50-16.00) | -3.50 (-23.00-9.00) | 12.50 (-6.25-47.25) | -5.50 (-11.13-1.63) | 4.50 (-6.88-33.00) | **0.005**  **0.008^a^**  **0.041^d^** |
|  | Subjective Total Sleep Time (PSQI Item 4 score) [hours] | 6.00 (5.38 - 7.00) | 6.50 (6.00-7.00) | 5.50 (4.25-6.00) | 6.50 (6.00-8.00) | 5.50 (5.00-6.50) | **0.003**  **0.006^a^** | 6.00 (5.00-7.00) | 6.00 (5.50-7.50) | 5.50 (4.75-6.50) | 6.50 (5.75-7.00) | 5.50 (4.63-6.38) | **0.007**  **0.005^a^ 0.016^c^ 0.028^d^ 0.047^f^** |
|  | Subjective to Objective Total Sleep Time Ratio [%] | 97.39 (81.39 - 119.76) | 111.11 (96.15-130.43) | 93.22 (68.74-101.77) | 105.26 (88.24-142.86) | 83.33 (68.97-93.70) | **0.005**  **0019^c^** | 95.19 (82.19-118.98) | 103.77 (92.19-133.11) | 87.77 (71.68-102.03) | 105.81 (87.68-125.03) | 81.47 (72.17-97.94) | **<0.001**  **0.008^a^ 0.002^c^ 0.022^d^ 0.031^f^** |
|  | BDI score | 12.50 (7.50 - 17.25) | 9.00 (4.00-14.00) | 15.00 (8.50-21.50) | 10.00 (5.00-15.00) | 22.00 (13.00-25.00) | **0.007**  **0.011^c^** | 10.00 (5.00-14.00) | 7.00 (4.00-12.00) | 13.00 (7.75-20.25) | 9.00 (4.75-13.00) | 14.00 (8.50-17.75) | **<0.001**  **<0.001^a^ <0.001^c^ 0.015^d^ 0.017^f^** |
|  | BDI score $\geq$14 | 23 (42.6%) | 6 (26.1%) | 7 (53.8%) | 2 (28.6%) | 8 (72.7%) | **0.048**  **0.023^c^** | 38 (29.9%) | 8 (14.5%) | 14 (46.7%) | 4 (18.2%) | 12 (60%) | **<0.001**  **0.002^a^**  **<0.001^c^**  **0.042^d^**  **0.010^f^** |
|  | BDI score $\geq$20 | 11 (20.4%) | 1 (4.3%) | 4 (30.8%) | 0 (0%) | 6 (54.5%) | **0.003**  **0.047^a^**  **0.002^c^**  **0.038^f^** | 14(11%) | 2 (3.6%) | 8 (26.7%) | 0 (0%) | 4(20%) | **0.002**  **0.003^a^**  **0.040^c^**  **0.015^d^**  **0.043^f^** |
|  | BDI score $\geq$29 | 3 (5.6%) | 1 (4.3%) | 2 (15.4%) | 0 (0%) | 0 (0%) | 0.319 | 4 (3.1%) | 0 (0%) | 1 (3.3%) | 0 (0%) | 3(15%) | **0.008**  **0.017^c^**  **0.049^f^** |

p-value for following comparisons: * Asymptomatic, Insomnia, Sleepiness, and Insomnia + Sleepiness groups, ^a^ Asymptomatic vs. Insomnia Group, ^b^ Asymptomatic vs. Sleepiness Group, ^c^ Asymptomatic vs. Insomnia + Sleepiness Group, ^d^ Insomnia vs. Sleepiness Group, ^e^ Insomnia vs. Insomnia + Sleepiness Group, ^f^ Sleepiness vs. Insomnia + Sleepiness Group

Abbreviations: AHI – apnea-hypopnea index; BDI – Beck Depression Index; BMI – body mass index; ESS – Epworth Sleepiness Scale; ISI – Insomnia Severity Scale; NREM – non-rapid eye movement; PSQI – Pittsburgh Sleep Quality Index; REM – rapid eye movement; SpO_2_ – oxygen saturation index; TST – total sleep time.
