# Supplementary material for: Evaluation of daytime sleepiness and insomnia symptoms in OSA patients with a characterization of symptom-defined phenotypes and their involvement in depression comorbidity—a cross-sectional clinical study
Source: Front Psychiatry. 2024 Mar 1;15:1303778. doi: 10.3389/fpsyt.2024.1303778 (PMC10940440; doi:10.3389/fpsyt.2024.1303778)
Supplement: Supplementary file 2 [file Table_2.docx]

Supplementary Material

Evaluation of daytime sleepiness and insomnia symptoms in OSA patients with a characterization of symptom-defined phenotypes and their involvement in depression comorbidity – a cross-sectional clinical study

**Agata Gabryelska*, Szymon Turkiewicz, Piotr Białasiewicz, Filip Grzybowski, Dominik Strzelecki, Marcin Sochal**

*** Correspondence:** Agata Gabryelska, MD, PhD; Department of Sleep Medicine and Metabolic Disorders at the Medical University of Lodz, 6/8 Mazowiecka Street, 90-419 Lodz, Poland, tel. +48 422725660, e-mail: agata.gabryelska@gmail.com

# Supplementary Table 2: Baseline characteristics and comparisons between distinct OSA phenotypes within severity groups.

|  | | Mild OSA Group | | | | | | Moderate OSA Group | | | | | | Severe OSA Group | | | | | |
| --- | --- | --- | --- | --- | --- | --- | --- | --- | --- | --- | --- | --- | --- | --- | --- | --- | --- | --- | --- |
| Phenotype | | Full Group | Asymptomatic | Insomnia | Sleepiness | Insomnia +Sleepiness | p-value* | Full Group | Asymptomatic | Insomnia | Sleepiness | Insomnia +Sleepiness | p-value* | Full Group | Asymptomatic | Insomnia | Sleepiness | Insomnia +Sleepiness | p-value* |
| Demographic Data | N | 42 | 19 | 10 | 7 | 6 | N/A | 29 | 16 | 5 | 5 | 3 | N/A | 56 | 20 | 15 | 10 | 11 | N/A |
|  | Age [years] | 52.69 +/- 12.14 | 53.89 +/- 13.12 | 50.30 +/- 11.06 | 57.00 +/- 10.41 | 47.83 +/- 12.92 | 0.456 | 54.93 +/- 11.09 | 56.88 +/- 11.72 | 51.40 +/- 10.02 | 57.40 +/- 9.94 | 46.33 +/- 10.26 | 0.366 | 54.70 +/- 11.25 | 56.45 +/- 10.65 | 55.60 +/- 9.80 | 55.10 +/- 13.78 | 49.91 +/- 11.96 | 0.562 |
|  | BMI [kg/m2] | 28.68 (26.38-31.00) | 29.11 (26.14-31.57) | 29.40 (25.51-33.03) | 26.86 (25.86-29.07) | 29.50 (25.97-29.64) | 0.684 | 30.99 (26.70-36.81) | 31.29 (25.83-36.81) | 28.77 (26.89-35.27) | 30.86 (27.38-36.88) | 38.20 (28.91-43.33) | 0.549 | 33.51 (29.61-38.74) | 32.19 (29.30-39.82) | 33.51 (28.31-38.74) | 36.07 (32.32-38.19) | 34.82 (30.92-42.41) | 0.585 |
|  | Sex (M) | 34 (81.0%) | 18 (94.7%) | 7 (70.0%) | 5 (71.4%) | 4 (66.7%) | 0.228 | 24 (82,8%) | 13 (81.3%) | 3 (60.0%) | 5 (100.0%) | 3 (100.0%) | 0.320 | 48 (85.7%) | 17 (85.0%) | 14 (93.3%) | 10 (100.0%) | 7 (63.6%) | 0.080 |
| PSG Data | Sleep Efficiency [%] | 79.75 (67.35-87.35) | 75.00 (65.00-88.75) | 84.90 (72.30-90.85) | 70.00 (56.05-77.50) | 83.50 (80.20-91.25) | 0.181 | 87.60 (80.30-91.90) | 86.10 (76.18-90.75) | 86.35 (85.10-91.85) | 90.40 (81.38-95.38) | 93.10 (86.20-95.00) | 0.460 | 82.70 (71.90-89.70) | 78.95 (68.90-85.70) | 85.35 (63.53-96.63) | 78.70 (53.43-86.65) | 90.40 (83.25-93.20) | 0.204 |
|  | Sleep Onset Latency [min] | 23.00 (11.75-41.25) | 31.00 (13.50-43.50) | 14.00 (7.25-23.13) | 23.00 (15.00-41.50) | 14.00 (6.25-36.25) | 0.064 | 21.00 (9.75-29.50) | 20.50 (12.63-33.13) | 28.50 (10.25-31.50) | 26.00 (8.75-27.25) | 29.50 (9.75-31.75) | 0.913 | 16.75 (8.75-30.13) | 17.75 (11.50-38.00) | 15.50 (7.50-33.00) | 17.25 (10.38-24.00) | 9..50 (7.00-26.00) | 0.672 |
|  | Sleep Maintenance Efficency [%] | 85.55 (75.08-93.75) | 87.80 (74.25-94.60) | 89.00 (77.20-94.40) | 76.40 (59.80-91.25) | 86.10 (85.00-92.00) | 0.584 | 92.00 (89.80-95.40) | 91.45 (84.35-94.43) | 91.40 (89.80-95.55) | 94.55 (86.90-97.63) | 93.40 (85.00-95.40) | 0.535 | 87.70 (79.95-92.95) | 86.10 (78.30-91.40) | 90.90 (68.50-97.90) | 83.55 (58.45-91.48) | 91.70 (87.25-95.20) | 0.413 |
|  | REM Sleep Latency [min] | 90.00 (71.50-148.30) | 90.00 (71.00-131.00) | 79.00 (66.50-145.70) | 126.50 (74.50-208.50) | 103.00 (62.25-136.75) | 0.611 | 78.50 (61.25-93.50) | 75.75 (59.13-91.25) | 88.00 (68.00-93.50) | 100.50 (59.75-125.25) | 87.75 (76.50-94.25) | 0.671 | 102.50 (65.50-169.88) | 118.00 (66.38-172.13) | 100.00 (67.00-171.00) | 94.75 (45.50-161.00) | 94.50 (75.00-198.00) | 0.847 |
|  | Total Sleep Time (TST) [hours] | 6.32 (5.28-6.90) | 6.34 (5.30-6.51) | 6.49 (5.52-7.09) | 5.20 (4.80-5.95) | 7.20 (6.24-8.03) | 0.061 | 6.70 (5.93-7.17) | 6.08 (5.63-6.79) | 7.60 (6.54-8.06) | 7.08 (6.45-7.80) | 5.80 (5.20-6.40) | 0.050 | 6.35 (5.03-6.98) | 5.75 (4.91-6.68) | 6.10 (4.70-6.50) | 6.54 (4.99-7.27) | 7.05 (6.10-7.70) | 0.064 |
|  | REM Percentage of TST [%] | 20.50 (15.55-27.50) | 19.00 (15.7-25.10) | 20.50 (12.80-28.75) | 21.60 (11.10-30.50) | 22.90 (19.70-27.85) | 0.711 | 22.00 (17.65-28.10) | 21.75 (17.08-28.08) | 24.50 (21.95-31.80) | 26.80 (14.05-32.75) | 19.70 (14.70-39.50) | 0.495 | 18.80 (12.40-23.95) | 17.00 (10.98-21.40) | 20.50 (15.10-24.00) | 20.60 (10.93-27.30) | 18.80 (13.30-24.60) | 0.403 |
|  | Stage 1 Percentage of TST [%] | 21.60 (14.05-31.05) | 27.50 (16.60-32.30) | 13.00 (4.28-30.20) | 24.40 (13.60-32.40) | 23.50 (19.15-24.80) | 0.277 | 21.30 (17.80-37.80) | 23.65 (15.48-39.20) | 20.80 (18.35-35.20) | 18.60 (12.70-53.85) | 24.00 (14.90-49.50) | 0.982 | 42.80 (26.68-56.68) | 37.45 (11.18-57.43) | 43.00 (38.40-54.40) | 54.50 (37.45-78.00) | 38.10 (20.90-50.50) | 0.198 |
|  | Stage 2 Percentage of TST [%] | 38.90 (32.15 - 47.95) | 36.70 (34.50 - 50.60) | 44.50 (38.03 - 49.68) | 38.90 (21.70 - 45.00) | 32.00 (30.40 - 44.05) | 0.466 | 34.30 (26.40 - 42.45) | 33.40 (27.00 - 46.30) | 32.30 (24.60 - 35.50) | 35.00 (12.15 - 47.80) | 35.00 (25.80 - 42.80) | 0.874 | 27.90 (17.85 - 36.45) | 30.65 (26.58 - 48.03) | 24.70 (20.30 - 31.80) | 17.25 (12.53 - 27.05) | 31.80 (15.20 - 36.80) | **0.029**  **0.026^b^** |
|  | Stage 3 Percentage of TST [%] | 16.30 (11.25-23.35) | 13.80 (10.30-18.10) | 21.60 (13.38-30.00) | 22.30 (1.10-27.60) | 16.30 (12.75-28.30) | 0.245 | 16.20 (10.05-22.65) | 16.75 (8.55-23.98) | 22.50 (8.35-24.20) | 15.80 (5.65-23.35) | 12.40 (8.80-24.60) | 0.898 | 9.05 (2.13-16.58) | 9.60 (2.25-21.20) | 8.90 (3.00-13.30) | 4.10 (0.00-13.60) | 13.90 (2.10-21.60) | 0.355 |
|  | REM Sleep Time [hours] | 1.22 (0.84 - 1.64) | 1.15 (0.86 - 1.63) | 1.06 (0.82 - 1.63) | 1.29 (0.63 - 1.58) | 1.55 (0.76 - 1.80) | 0.837 | 1.40 (1.05 - 1.99) | 1.35 (0.93 - 1.85) | 2.06 (1.44 - 2.43) | 1.80 (0.94 - 2.56) | 1.37 (0.76 - 2.88) | 0.292 | 1.05 (0.73 - 1.48) | 0.98 (0.54 - 1.28) | 1.03 (0.79 - 1.54) | 1.22 (0.71 - 1.90) | 1.17 (0.82 - 1.88) | 0.473 |
|  | Stage 1 Time [hours] | 1.31 (0.87-1.83) | 1.41 (1.11-2.02) | 0.89 (0.31-1.38) | 1.40 (0.71-1.93) | 1.43 (0.96-1.60) | 0.201 | 1.47 (1.07-2.21) | 1.45 (1.02-2.15) | 1.75 (1.20-2.70) | 1.43 (0.97-3.41) | 1.67 (0.86-3.51) | 0.944 | 2.35 (1.57-3.37) | 2.24 (0.66-2.85) | 2.51 (2.13-3.01) | 3.24 (2.06-4.22) | 1.93 (1.35-3.51) | 0.195 |
|  | Stage 2 Time [hours] | 2.34 (2.12 - 2.97) | 2.32 (2.19 - 3.20) | 2.77 (2.48 - 3.42) | 2.02 (1.54 - 2.68) | 2.25 (1.18 - 2.93) | 0.188 | 2.27 (1.47 - 2.79) | 2.14 (1.50 - 3.04) | 2.27 (1.76 - 2.67) | 2.70 (0.80 - 3.53) | 2.03 (1.34 - 2.73) | 0.991 | 1.59 (1.06 - 2.23) | 1.83 (0.92 - 3.13) | 1.52 (1.31 - 1.81) | 1.14 (0.78 - 1.45) | 1.79 (1.00 - 2.83) | 0.062 |
|  | Stage 3 Time [hours] | 1.02 (0.69 - 1.36) | 0.83 (0.67 - 1.09) | 1.33 (0.78 - 1.69) | 1.08 (0.07 - 1.58) | 1.13 (0.57 - 1.52) | 0.243 | 1.08 (0.58 - 1.54) | 1.06 (0.52 - 1.50) | 1.52 (0.64 - 1.76) | 1.19 (0.45 - 1.61) | 0.86 (0.46 - 1.61) | 0.815 | 0.51 (0.14 - 1.08) | 0.48 (0.15 - 1.18) | 0.62 (0.14 - 0.80) | 0.25 (0.00 - 0.80) | 0.84 (0.16 - 1.81) | 0.294 |
|  | NREM Sleep Time [hours] | 4.93 (4.27-5.34) | 5.04 (4.27-5.28) | 4.89 (4.32-5.78) | 4.62 (3.23-4.93) | 5.25 (4.78-6.89) | 0.221 | 4.90 (4.62-5.46) | 4.80 (4.54-4.95) | 5.48 (4.79-5.97) | 5.34 (5.02-5.70) | 4.44 (4.21-5.40) | 0.070 | 4.98 (4.07-5.64) | 4.92 (4.07-5.57) | 4.77 (4.04-5.09) | 4.96 (3.92-5.93) | 5.43 (4.78-6.50) | 0.230 |
|  | NREM Percentage of TST [%] | 79.80 (73.10-84.63) | 81.00 (74.90-84.30) | 79.50 (71.25-87.20) | 78.40 (69.50-88.90) | 78.60 (74.13-85.38) | 0.946 | 78.00 (71.90-82.35) | 78.25 (71.93-82.93) | 75.50 (68.20-78.05) | 73.20 (67.25-85.95) | 80.30 (72.50-86.70) | 0.495 | 81.20 (76.05-87.60) | 83.00 (78.60-89.03) | 79.50 (76.00-84.90) | 79.40 (72.70-89.08) | 81.20 (75.40-86.70) | 0.403 |
|  | Arousal Index [events/hour] | 11.0 (7.20-17.40) | 15.10 (8.60-18.70) | 8.10 (6.10-13.73) | 10.20 (6.58-17.70) | 11.70 (9.00-15.35) | 0.527 | 14.90 (10.60-20.90) | 15.05 (10.80-20.13) | 12.50 (8.70-23.25) | 14.60 (9.70-27.95) | 24.40 (8.20-31.40) | 0.855 | 24.25 (17.78-34.35) | 23.25 (17.85-33.30) | 26.90 (18.60-34.40) | 32.80 (21.48-40.85) | 21.70 (14.10-34.90) | 0.299 |
|  | AHI in REM [events/hour] | 9.49 (5.29-15.97) | 7.88 (5.48-9.81) | 9.87 (3.96-15.35) | 14.78 (5.00-29.13) | 9.91 (5.40-27.87) | 0.430 | 25.60 (13.81-33.31) | 25.88 (13.08-31.88) | 37.48 (13.72-47.95) | 24.62 (6.20-42.63) | 25.60 (20.69-28.48) | 0.772 | 49.59 (30.95-65.77) | 49.59 (25.03-63.99) | 46.12 (30.95-68.94) | 50.66 (36.74-66.97) | 46.27 (30.70-69.68) | 0.922 |
|  | AHI in NREM [events/hour] | 7.49 (4.70-9.98) | 6.47 (4.95-8.37) | 8.33 (4.36-11.08) | 9.80 (4.04-15.46) | 6.38 (2.88-14.27) | 0.511 | 19.19 (15.65-20.89) | 18.99 (15.57-21.72) | 19.19 (16.43-20.50) | 15.61 (13.28-20.00) | 27.45 (17.84-29.19) | 0.287 | 45.93 (36.05-66.95) | 42.51 (34.85-59.09) | 40.71 (35.70-76.39) | 50.60 (36.74-66.97) | 47.64-43.83-71.39) | 0.525 |
|  | AHI [events/hour] | 8.95 (6.98-12.08) | 7.60 (5.60-9.50) | 8.60 (7.53-11.10) | 12.90 (10.50-13.60) | 11.45 (8.45-13.20) | **0.007**  **0.012^b^** | 20.30 (18.05-25.75) | 19.95 (17.65 -22.83) | 19.80 (18.35-28.30) | 23.10 (17.60-25.00) | 27.80 (19.10-34.50) | 0.447 | 47.65 (38.93-68.95) | 46.90 (39.33-54.90) | 51.50 (36.00-74.10) | 53.70 (36.28-71.63) | 51.40 (40.80-74.10) | 0.738 |
|  | Total Number of Desaturations | 56.50 (33.0-74.25) | 55.00 (28.00-65.50) | 48.00 (32.00-100.50) | 63.00 (47.50-68.00) | 86.00 (84.00-90.50) | 0.071 | 131.00 (110.00-178.00) | 130.00 (111.00-143.50) | 166(107.00-211.50) | 190.00 (142.75-303.25) | 105.00 (105.00-371.50) | 0.137 | 304.00 (208.00-504.50) | 277.50 (202.50-421.50) | 276.00 (209.50-401.00) | 329.50 (207.50-466.50) | 377.00 (188.50-678.50) | 0.960 |
|  | Desaturation Index [events/hour] | 8.35 (6.00-11.78) | 8.00 (5.60-9.00) | 8.50 (7.50-11.40) | 11.20 (7.40-14.00) | 12.50 (10.48-13.65) | **0.009**  **0.011^c^** | 21.70 (18.75-27.25) | 20.75 (18.63-24.45) | 20.10 (16.80-30.65) | 26.00 (19.90-40.50) | 29.60 (18.00-43.10) | 0.470 | 51.80 (37.15-70.93) | 47.90 (34.70-65.65) | 51.00 (37.00-73.00) | 60.50 (39.13-72.88) | 53.20 (44.70-83.60) | 0.684 |
|  | Basal SpO_2_ [%] | 93.30 (92.25-94.00) | 93.60 (92.60-95.00) | 92.50 (91.80-94.00) | 93.00 (92.70-94.10) | 92.40 (91.05-95.00) | 0.290 | 92.70 (92.00-94.00) | 92.60 (92.00-94.00) | 93.90 (92.00-94.25) | 92.50 (90.50-93.85) | 92.00 (92.00-94.00) | 0.793 | 92.00 (88.95-93.00) | 92.25 (90.85-93.15) | 91 (87.00-93.00) | 90.50 (86.98-91.75) | 92.00 (82.00-94.00) | 0.445 |
|  | Mean SpO_2_ during desaturations, [%] | 89.70 (88.00-91.40) | 90.20 (89.00-92.00) | 88.10 (87.00-91.00) | 89.00 (88.10-91.40) | 88.75 (85.30-93.75) | 0.194 | 88.80 (87.55-90.00) | 88.50 (87.33-89.90) | 89.00 (88.50-90.60) | 88.50 (86.35-90.75) | 89.00 (86.00-90.00) | 0.611 | 86.20 (83.25-89.00) | 88.15 (85.25-89.00) | 86.90 (81.60-88.00) | 82.50 (78.88-85.58) | 87.00 (75.00-90.00) | 0.076 |
|  | Minimum SpO2 [%] | 84.00 (81.60-87.00) | 86.00 (83.00-88.80) | 82.05 (77.00-85.73) | 84.00 (81.90-86.00) | 81.90 (61.65-88.00) | 0.236 | 79.00 (75.10-82.00) | 78.00 (74.30-82.90) | 79.00 (77.00-82.00) | 73.25 (17.53-79.10) | 81.90 (80.00-82.23) | 0.295 | 74.90 (65.50-80.65) | 79.00 (68.75-80.55) | 74.00 (58.70-79.70) | 67.55 (63.75-72.75) | 75.00 (55.28-82.23) | 0.336 |
| Questionaire Data | ESS score | 8.50 (5.00-11.25) | 5.00 (3.00-8.00) | 8.00 (4.00-10.00) | 12.00 (11.00-14.00) | 14.50 (12.00-17.50) | **<0.001**  **0.001^b^ <0.0001^c^ 0.036^d^ 0.009^e^** | 8.00 (5.00-11.00) | 5.00 (3.25-7.00) | 8.00 (7.00-8.50) | 13.00 (12.00-18.50) | 11.00 (11.00-18.00) | **<0.001**  **0.001^b^ 0..039^c^ 0.048^d^** | 8.00 (5.00-12.75) | 6.00 (3.00-8.00) | 5.00 (4.00-8.00) | 12.50 (11.00-13.00) | 16.00 (17.00-20.00) | **<0.001**  **0.001^b^ <0.0001^c^ 0.002^d^ <0.0001^e^** |
|  | ESS score $\geq$11 | 13 (31%) | 0 (0%) | 0 (0%) | 7 (100%) | 6 (100%) | **<0.001**  **<0.001^b^**  **<0.001^c^**  **<0.001^d^**  **<0.001e** | 8 (27.6%) | 0 (0%) | 0 (0%) | 5 (100%) | 3 (100%) | **<0.001**  **<0.001^b^**  **<0.001^c^**  **<0.001^d^**  **<0.001^e^** | 21 (37.5%) | 0 (0%) | 0 (0%) | 10 (100%) | 11 (100%) | <0.001  <0.001^b^  <0.001^c^  <0.001^d^  <0.001^e^ |
|  | ISI score | 12.00 (9.00-17.00) | 10.00 (8.00-12.00) | 17.00 (16.00-20.25) | 9.00 (8.00-11.00) | 17.00 (15.00-18.25) | **<0.001**  **<0.0001^a^ 0.005^c^ <0.000^d^ 0.006^f^** | 11.00 (8.50-15.00) | 10.00 (7.25-12.00) | 17.00 (15.00-22.50) | 10.00 (7.00-12.00) | 18.00 (15.00-20.00) | **<0.001**  **0.005 ^a^ 0.047^c^ 0.031^d^ 0.045^f^** | 14.00 (9.00-17.00) | 8.50 (7.00-11.75) | 17.00 (16.00-19.00) | 11.50 (8.75-13.25) | 17.00 (16.00-20.00) | **<0.001**  **<0.0001^a^ <0.0001^c^ 0.002^d^ 0.003^f^** |
|  | ISI score$\geq$15 | 16 (38.1%) | 0(0%) | 10 (100%) | 0 (0%) | 6 (100%) | **<0.001**  **<0.001^a^**  **<0.001^c^**  **<0.001^d^**  **<0.001^f^** | 8 (27.6%) | 0 (0%) | 0 (0%) | 5 (100%) | 3 (100%) | **<0.001**  **<0.001^a^**  **<0.001^c^**  **<0.001^d^**  **<0.001^f^** | 26 (46.4%) | 0(0%) | 15 (100%) | 0 (0%) | 11 (100%) | <0.001  <0.001^a^  <0.001^c^  <0.001^d^  <0.001^f^ |
|  | Subjective Sleep Latency (PSQI Item 2) [minutes] | 18.75 (6.88-41.25) | 15.00 (5.00-30.00) | 35.00 (16.88-82.50) | 17.50 (5.00-45.00) | 17.50 (3.00-52.50) | 0.187 | 30.00 (11.25-36.25) | 30.00 (10.63-35.00) | 45.00 (25.00-90.00) | 7.50 (4.00-15.00) | 30.00 (12.50-34.00) | **0.028**  **0.018^d^** | 15.00 (8.13-33.75) | 15.00 (8.13-30.00) | 20.00 (15.00-45.00) | 12.50 (7.13-20.75) | 15.00 (5.50-35.00) | 0.346 |
|  | Subjective to Objective Sleep Latency Ratio [%] | 68.38 (36.65-183.69) | 60.00 (24.39-100.00) | 363.64 (103.83-664.71) | 55.56 (43.48-133.58) | 58.82 (35.71-281.51) | 0.010  0.005a | 138.89 (52.18-272.22) | 120.92 (52.68-238.33) | 266.67 (157.89-346.99) | 52.63 (15.38-133.22) | 300.00 (48.80-450.11) | 0.085 | 94.80 (50.00-229.90) | 94.80 (40.58-211.27) | 193.55 (49.18-413.79) | 63.16 (58.40-117.17) | 113.21 (25.00-230.77) | 0.547 |
|  | Difference between Subjective and Objective Sleep Latency [min] | -4.50 (-13.63-20.88) | -11.50 (-31.00- 0.00) | 17.50 (1.63-73.13) | -6.50 (-13.00-18.50) | 5.25 (-10.63-40.75) | **0.008**  **0.007^a^** | 3.00 (-12.00-21.50) | 2.00 (-9.75-19.00) | 31.50 (7.00-58.75) | -13.50 (-22.00-2.50) | 20.00 (-54.00-34.00) | 0.086 | -0.50 (-9.75-14.50) | -0.50 (-20.25-13.00) | 5.00 (-15.00-29.00) | -5.00 (-8.13-1.63) | 3.50 (-6.50-34.00) | 0.633 |
|  | Subjective Total Sleep Time (PSQI Item 4 score) [hours] | 6.00 (5.50-7.00) | 6.00 (5.50-7.00) | 5.50 (4.63-7.00) | 6.50 (5.00-7.00) | 6.00 (5.50-7.63) | 0.773 | 7.00 (5.50-8.00) | 7.00 (6.00-8.00) | 5.50 (4.00-6.50) | 7.00 (6.75-8.25) | 5.00 (4.20-6.50) | 0.054 | 6.00 (5.00-6.50) | 6.00 (5.00-7.38) | 6.00 (5.00-6.00) | 6.25 (4.88-7.13) | 5.00 (4.50-6.50) | 0.298 |
|  | Subjective to Objective Total Sleep Time Ratio [%] | 95.38 (81.19-124.87) | 94.61 (87.84-140.35) | 89.30 (71.68-102.03) | 113.41 (96.15-135.42) | 90.39 (73.74-115.70) | 0.245 | 101.45 (80.00-121.39) | 109.98 (99.09-132.24) | 65.35 (53.93-98.46) | 104.48 (90.56-118.14) | 80.74 (79.25-92.80) | **0.024**  **0.039^a^** | 93.24 (83.38-117.41) | 99.96 (90.99-126.99) | 91.55 (81.97-120.00) | 103.91 (82.24-121.74) | 77.38 (65.57-94.20) | **0.028**  **0.018^c^** |
|  | BDI score | 10.00 (5.00-14.00) | 10.00 (5.00-13.00) | 13.00 (11.50-15.50) | 5.00 (3.00-8.00) | 15.50 (6.75-18.50) | **0.013**  **0.034^d^ 0.048^f^** | 11.00 (5.00-14.00) | 7.00 (4.00-12.75) | 14.00 (8.50-21.00) | 10.00 (2.50-13.00) | 14.00 (14.00-31.00) | **0.045**  **0.047^a^**  **0.042^c^**  **0.036^f^** | 10.00 (6.00-15.00) | 6.00 (2.00-10.50) | 12.00 (7.00-22.00) | 12.50 (8.00-15.25) | 10.00 (8.00-32.00) | **0.003**  **0.012^a^ 0.025^c^** |
|  | BDI score $\geq$14 | 11 (26.2%) | 3 (15.8%) | 4 (40%) | 0 (0%) | 4 (66.7%) | **0.022**  **0.032^c^**  **0.021^f^** | 9 (31%) | 3 (18.8%) | 3 (60%) | 0(0%) | 3 (100%) | **0.007**  **0.021^c^**  **0.018^f^** | 18(32.1%) | 2 (10%) | 7 (46.7%) | 4 (40%) | 5 (45.5%) | **0.048**  **0.022^a^**  **0.037^c^** |
|  | BDI score $\geq$20 | 5 (11.9%) | 2 (10.5%) | 2 (20%) | 0 (0%) | 1 (16.7%) | 0.629 | 1 (3.4%) | 0 (0%) | 1(20%) | 0(0%) | 0 (0%) | 0.174 | 8 (14.3%) | 0(0%) | 5 (33.3%) | 0(0%) | 3 (27.3%) | **0.012**  **0.009^a^**  **0.037^c^** |
|  | BDI score $\geq$29 | 0 (0%) | 0 (0%) | 0(0%) | 0(0%) | 0 (0%) | N/A | 0 (0%) | 0 (0%) | 0(0%) | 0(0%) | 0(0% | N/A | 4 (7.1%) | 0(0%) | 1 (6.7%) | 0(0%) | 3 (27.3%) | **0.029**  **0.037^c^** |

*P*-value for following comparisons: * Asymptomatic, Insomnia, Sleepiness, and Insomnia + Sleepiness groups, ^a^ Asymptomatic vs. Insomnia Group, ^b^ Asymptomatic vs. Sleepiness Group, ^c^ Asymptomatic vs. Insomnia + Sleepiness Group, ^d^ Insomnia vs. Sleepiness Group, ^e^ Insomnia vs. Insomnia + Sleepiness Group, ^f^ Sleepiness vs. Insomnia + Sleepiness Group

Abbreviations: AHI – apnea-hypopnea index; BDI – Beck Depression Index; BMI – body mass index; ESS – Epworth Sleepiness Scale; ISI – Insomnia Severity Scale; NREM – non-rapid eye movement; PSQI – Pittsburgh Sleep Quality Index; REM – rapid eye movement; SpO_2_ – oxygen saturation
