# Supplementary material for: Evaluation of daytime sleepiness and insomnia symptoms in OSA patients with a characterization of symptom-defined phenotypes and their involvement in depression comorbidity—a cross-sectional clinical study
Source: Front Psychiatry. 2024 Mar 1;15:1303778. doi: 10.3389/fpsyt.2024.1303778 (PMC10940440; doi:10.3389/fpsyt.2024.1303778)
Supplement: Supplementary file 3 [file Table_3.docx]

Supplementary Material

Evaluation of daytime sleepiness and insomnia symptoms in OSA patients with a characterization of symptom-defined phenotypes and their involvement in depression comorbidity – a cross-sectional clinical study

**Agata Gabryelska*, Szymon Turkiewicz, Piotr Białasiewicz, Filip Grzybowski, Dominik Strzelecki, Marcin Sochal**

*** Correspondence:** Agata Gabryelska, MD, PhD; Department of Sleep Medicine and Metabolic Disorders at the Medical University of Lodz, 6/8 Mazowiecka Street, 90-419 Lodz, Poland, tel. +48 422725660, e-mail: agata.gabryelska@gmail.com

# Supplementary Table 3: Comparison of phenotypes between control and OSA groups.

|  | | Comparisons between control and OSA groups | | | | Comparisons between control and OSA severity groups | | | | |
| --- | --- | --- | --- | --- | --- | --- | --- | --- | --- | --- |
| Phenotype | | Asymptomatic | Insomnia | Sleepiness | Insomnia + Sleepiness | Asymptomatic | Insomnia | Sleepiness | Insomnia +Sleepiness |  |
| Demographic Data | Age [years] | **0.018** | 0.214 | **0.005** | 0.090 | 0.093 | 0.359 | 0.053 | 0.373 |  |
|  | BMI [kg/m2] | **0.019** | 0.204 | 0.285 | **0.017** | **0.023**  **0.021^c^** | 0.193 | **0.011**  **0.009^e^** | **0.022**  **0.031^c^** |  |
|  | Sex (M) | **0.032** | 0.137 | 0.238 | 0.873 | 0.102 | 0.117 | 0.174 | 0.663 |  |
| PSG Data | Sleep Efficiency [%] | 0.889 | 0.162 | 0.122 | 0.560 | 0.551 | 0.525 | 0.059 | 0.648 |  |
|  | Sleep Onset Latency [min] | 0.610 | 0.014 | 0.460 | 0.863 | 0.283 | 0.075 | 0.674 | 0.999 |  |
|  | Sleep Maintenance Efficency [%] | 0.512 | 0.427 | 0.428 | 0.634 | 0.612 | 0.851 | 0.281 | 0.630 |  |
|  | REM Sleep Latency [min] | 0.705 | 0.135 | 0.646 | 0.507 | 0.230 | 0.337 | 0.539 | 0.597 |  |
|  | Total Sleep Time (TST) [hours] | 0.939 | 0.508 | 0.592 | 0.836 | 0.738 | 0.067 | 0.140 | 0.410 |  |
|  | REM Percentage of TST [%] | 0.110 | 0.128 | 0.838 | 0.712 | **0.036**  **0.049^c^** | 0.118 | 0.772 | 0.587 |  |
|  | Stage 1 Percentage of TST [%] | **<0.001** | **0.017** | **0.047** | **0.005** | **0.003**  **0.001^c^** | **<0.001**  **<0.0001^c^**  **<0.0001^e^** | **0.011**  **0.015^c^** | **0.044**  **0.042^c^** |  |
|  | Stage 2 Percentage of TST [%] | **0.045** | **<0.001** | **0.023** | **0.022** | 0.086 | **<0.001**  **<0.0001^c^**  **0.020^e^** | **0.007**  **0.008^c^** | 0.121 |  |
|  | Stage 3 Percentage of TST [%] | **0.011** | 0.570 | 0.102 | 0.301 | **0.024**  **0.019^c^** | **0.012**  **0.010^e^** | 0.058 | 0.525 |  |
|  | REM Sleep Time [hours] | 0.194 | 0.204 | 0.939 | 0.247 | **0.047**  **0.017^c^** | 0.067 | 0.650 | 0.668 |  |
|  | Stage 1 Time [hours] | **<0.001** | **0.010** | 0.067 | **0.004** | **0.004**  **0.003^c^** | **<0.001**  **<0.0001^c^**  **<0.0001^e^** | **0.016**  **0.027^c^** | **0.018**  **0.013^c^** |  |
|  | Stage 2 Time [hours] | 0.185 | **0.002** | **0.014** | 0.386 | 0.265 | **<0.001**  **<0.0001^c^**  **0.015^e^** | **0.008**  **0.006^c^** | 0.859 |  |
|  | Stage 3 Time [hours] | **0.026** | 0.791 | 0.102 | 0.967 | **0.041**  **0.040^c^** | **0.007**  **0.009^e^** | 0.056 | 0.983 |  |
|  | NREM Sleep Time [hours] | 0.518 | 0.543 | 0.838 | 0.536 | 0.936 | 0.306 | 0.189 | 0.589 |  |
|  | NREM Percentage of TST [%] | 0.110 | 0.128 | 0.838 | 0.363 | **0.036**  **0.049^c^** | 0.118 | 0.772 | 0.732 |  |
|  | Arousal Index [events/hour] | **<0.001** | **0.044** | 0.124 | **<0.001** | **<0.001**  **<0.0001^c^**  **0.003^e^**  **0.030^f^** | **<0.001**  **0.001^c^**  **0.002^e^** | **0.008**  **0.036^c^**  **0.021^e^** | **0.002**  **0.001^c^** |  |
|  | AHI in REM [events/hour] | **<0.001** | **<0.001** | **<0.001** | **<0.001** | **<0.001**  **<0.0001^b^**  **<0.0001^c^**  **<0.0001^e^** | **<0.001**  **<0.0001^c^**  **0.002^e^** | **0.001**  **<0.0001c** | **<0.001**  **<0.0001^c^** |  |
|  | AHI in NREM [events/hour] | **<0.001** | **<0.001** | **<0.001** | **<0.001** | **<0.001**  **<0.0001^b^**  **<0.0001^c^**  **<0.0001^e^** | **<0.001**  **<0.0001^c^**  **0.001^e^** | **<0.001**  **<0.0001^c^**  **0.011^e^** | **<0.001**  **<0.0001^c^**  **0.022^e^** |  |
|  | AHI [events/hour] | **<0.001** | **<0.001** | **<0.001** | **<0.001** | **<0.001**  **<0.0001^b^**  **<0.0001^c^**  **<0.0001^e^** | **<0.001**  **<0.0001^c^**  **0.002^e^** | **<0.001**  **<0.0001^c^**  **0.008^e^** | **<0.001**  **<0.0001^c^** |  |
|  | Total Number of Desaturations | **<0.001** | **0.003** | **0.002** | **0.004** | **<0.001**  **0.001^b^**  **<0.0001^c^**  **0.044^e^** | **0.002**  **0.002^c^**  **0.015^e^** | **0.002**  **0.019^c^**  **0.005^e^** | 0.018  0.011^c^ |  |
|  | Desaturation Index [events/hour] | **<0.001** | **<0.001** | **<0.001** | **<0.001** | **<0.001**  **<0.0001^b^**  **<0.0001^c^**  **<0.0001^e^** | **<0.001**  **0.017^b^**  **<0.0001^c^**  **0.004^e^** | **<0.001**  **<0.0001^c^**  **0.005^e^** | **<0.001**  **<0.0001^c^** |  |
|  | Basal SpO_2_ [%] | **0.029** | **0.011** | 0.059 | 0.093 | **0.005**  **0.013^c^**  **0.029^e^** | **0.013**  **0.008^c^** | **0.014**  **0.027^c^** | 0.280 |  |
|  | Mean SpO_2_ during desaturations, [%] | **0.007** | **0.002** | **0.019** | 0.090 | **<0.001**  **0.033^b^**  **0.001^c^**  **0.035^d^**  **0.002^e^** | **<0.001**  **<0.0001^c^** | **<0.001**  **0.002^c^**  **0.011^e^** | 0.232 |  |
|  | Minimum SpO2 [%] | **<0.001** | **0.023** | **0.012** | 0.090 | **<0.001**  **<0.0001^b^**  **<0.0001^c^**  **0.038^d^**  **0.001^e^** | **0.012**  **0.014^c^** | **0.001**  **0.007^c^**  **0.024^e^** | 0.303 |  |
| Questionaire Data | ESS score | 0.787 | 0.699 | 0.917 | 0.099 | 0.970 | 0.500 | 0.495 | 0.142 |  |
|  | ISI score | 0.434 | 0.904 | 0.095 | **0.004** | 0.750 | 0.987 | 0.151 | **0.025**  **0.035^a^** |  |
|  | Subjective Sleep Latency (PSQI Item 2) [minutes] | 0.555 | 0.313 | 0.837 | **0.006** | 0.360 | 0.342 | 0.669 | 0.051 |  |
|  | Subjective to Objective Sleep Latency Ratio [%] | 0.443 | 0.169 | 0.980 | **0.029** | 0.111 | 0.314 | 0.633 | 0.137 |  |
|  | Difference between Subjective and Objective Sleep Latency [min] | 0.210 | 0.321 | 0.939 | **0.010** | 0.058 | 0.273 | 0.708 | 0.084 |  |
|  | Subjective Total Sleep Time (PSQI Item 4 score) [hours] | 0.576 | 0.329 | 0.623 | 0.884 | 0.159 | 0.768 | 0.242 | 0.198 |  |
|  | Subjective to Objective Total Sleep Time Ratio [%] | 0.685 | 0.979 | 0.760 | 0.885 | 0.765 | 0.305 | 0.820 | 0.762 |  |
|  | BDI score | 0.351 | 0.569 | 0.645 | 0.116 | 0.320 | 0.941 | 0.070 | 0.476 |  |
|  | BDI score $\geq$14 | 0.186 | 0.460 | 0.455 | 0.381 | 0.581 | 0.868 | 0.129 | 0.292 |  |
|  | BDI score $\geq$20 | 0.655 | 0.529 | N/A | 0.060 | 0.290 | 0.864 | N/A | 0.193 |  |
|  | BDI score $\geq$29 | 0.295 | 0.213 | N/A | 0.254 | 0.489 | 0.468 | N/A | 0.110 |  |

p-value for following comparisons: * Control, Mild OSA, Moderate OSA and Sever OSA groups, a Control vs. Mild OSA Group, b Control vs. Moderate OSA Group, c Control vs. Severe OSA Group, d Mild OSA vs. Moderate OSA Group, e Mild OSA vs. Sever OSA Group, f Moderate OSA vs. Severe OSA Group.

Abbreviations: AHI – apnea-hypopnea index; BDI – Beck Depression Index; BMI – body mass index; ESS – Epworth Sleepiness Scale; ISI – Insomnia Severity Scale; NREM – non-rapid eye movement; PSQI – Pittsburgh Sleep Quality Index; REM – rapid eye movement; SpO2 – oxygen saturation index; TST – total sleep time.
